# Valuable Prognostic Role of Disability, Pain, Anxiety, and Depression Scales in Instrumented Lumbar Spine Surgery for Degenerative Pathology: The SAP-LD Study

**DOI:** 10.3390/brainsci15101035

**Published:** 2025-09-24

**Authors:** Anita Simonini, Pier Paolo Panciani, Riccardo Bergomi, Giorgio Saraceno, Carlo Brembilla, Gabriele Capo, Nicola Montemurro, Claudio Rossi, Edoardo Agosti, Linda Gritti, Gennaro Salierno, Marco Maria Fontanella, Luca Zanin

**Affiliations:** 1 Neurosurgery Unit, Department of Medical and Surgical Specialties, Radiological Sciences and Public Health, University of Brescia, 25121 Brescia, Italypierpaolo.panciani@unibs.it (P.P.P.);; 2 Unit of Neurosurgery, ASST Spedali Civili di Brescia, 25123 Brescia, Italy; 3 Department of Neurosurgery, IRCCS Humanitas Research Hospital, 20089 Rozzano, Italygabriele.capo@humanitas.it (G.C.); 4 Department of Neurosurgery, Azienda Ospedaliero Universitaria Pisana (AOUP), 56100 Pisa, Italy; nicola.montemurro@unipi.it; 5 Department of Statistics and Quantitative Methods, University of Milano-Bicocca, 20126 Milano, Italy

**Keywords:** degenerative lumbar spine disease, instrumented spine surgery, owestry disability index, pain catastrophizing scale, hospital anxiety and depression scale, SF-36 health survey, postoperative outcomes, psychological factors, quality of life, spine surgery prognosis

## Abstract

**Background:** Degenerative lumbar spine disease is a prevalent cause of chronic low back pain that significantly impairs daily function and quality of life. While conservative management is the first line of treatment, many patients ultimately require instrumented lumbar spine surgery. However, postoperative outcomes vary considerably, with emerging evidence suggesting that preoperative psychological factors such as anxiety, depression, and pain catastrophizing may influence recovery. The SAP-LD (Scale for Anxiety and Pain in Lumbar Degeneration) study was designed to assess the prognostic role of these psychological and physical parameters in surgical outcomes. **Methods:** This prospective observational study enrolled 70 adult patients with degenerative lumbar spine pathology scheduled for instrumented surgical treatment at the University of Brescia and ASST Spedali Civili di Brescia between March and December 2024. Preoperative assessments included demographic, clinical, and radiologic data along with validated scales: the Oswestry Disability Index (ODI), 36-Item Short Form Health Survey (SF-36), Visual Analogue Scale (VAS), Pain Catastrophizing Scale (PCS), and Hospital Anxiety and Depression Scale (HADS). Follow-up evaluations were performed at 45 days and at 6 months, and statistical analyses were conducted using correlation tests, ANOVA, and regression modeling. **Results:** The demographic analysis of the 70 enrolled patients shows a balanced gender distribution (38 females, 34 males) with a mean age of 61 years (range 23–81). The educational level distribution indicates that the majority of patients (44.29%) have a secondary education level, while 35.71% have a tertiary education level. Regarding employment status, 50% of the patients are retired or not working. Patients with clinically significant anxiety and/or depression showed higher levels of perceived pain, pain catastrophizing, and disability at baseline. These patients reported significantly worse scores on the Visual Analogue Scale (VAS), Pain Catastrophizing Scale (PCS), and Oswestry Disability Index (ODI). The Oswestry Disability Index (ODI) demonstrates a clinically significant improvement (reduction) in disability between the preoperative period (t0) and the 45-day follow-up (t2), with the median decreasing from 39.00 to 13.00. However, there is a partial regression at the 6-month follow-up (t3), with the median increasing to 27.00. For the SF-36 Health Survey, the General Health subscale shows an improvement between t0 and t2 (median increasing from 55.00 to 60.00), followed by a slight decrease at t3 (median 55.00). Similar patterns are observed in most other subscales, with initial improvement followed by partial regression. The Pain Catastrophizing Scale (PCS) shows a substantial reduction in catastrophizing between t0 and t2 (median decreasing from 16.00 to 3.00), followed by an increase at t3 (median 11.00), though still below baseline levels. Pain intensity as measured by the Visual Analogue Scale (VAS) shows a significant reduction at t2 (median decreasing from 5.00 to 3.00), but increases again at t3 (median 6.00), even exceeding the preoperative level. For the Hospital Anxiety and Depression Scale (HADS), no significant differences were observed across time points, with values indicating mild symptoms throughout the study period. Correlation analyses confirmed that higher preoperative anxiety and depression scores were predictive of poorer postoperative outcomes. Specifically, higher HADS scores at baseline are associated with higher ODI scores (increased disability) at all time points (*p* = 0.002), higher VAS scores (increased pain) at all time points (*p* = 0.015), and lower scores on SF-36 subscales, particularly Emotional Well-being (*p* = 0.00023) and Social Functioning (*p* = 0.002). Higher PCS scores at baseline are associated with higher ODI scores at all time points (*p* = 0.001), higher VAS scores at all time points (*p* = 0.008), and lower scores on SF-36 subscales, particularly Pain (*p* = 0.00023) and Physical Functioning (*p* = 0.04254). The mixed linear models analysis confirms these findings, showing that the ODI score decreases significantly between t0 and t2 (*p* = 0.00023) and increases between t2 and t3, though this increase is not statistically significant (*p* = 0.079). For VAS scores, there is a significant decrease between t0 and t2 (*p* = 0.00023) and a significant increase between t2 and t3 (*p* = 0.04254). Patients with elevated preoperative HADS scores tended to have slower recovery trajectories and reported lower satisfaction levels. These findings reinforce the prognostic value of psychological assessments in spine surgery and suggest that targeted psychological interventions could improve patient outcomes. **Conclusions:** By identifying psychological predictors of postoperative recovery, this study underscores the importance of integrating preoperative psychological screening into routine clinical practice. The results suggest that a multidisciplinary approach, including both surgical and psychological care, could enhance long-term functional outcomes and quality of life for patients undergoing instrumented lumbar spine surgery.

## 1. Introduction

Degenerative lumbar spine disease represents one of the most common causes of chronic low back pain, significantly affecting patients’ quality of life and functional capacity [1,2]. This condition encompasses a spectrum of pathologies including disk herniation, spinal stenosis, and degenerative spondylolisthesis, all of which can lead to persistent pain, neurological deficits, and progressive disability. While conservative management approaches—including physical therapy, pain medication, and lifestyle modifications—constitute the first line of treatment, a substantial proportion of patients ultimately require surgical intervention when non-operative measures fail to provide adequate symptom relief [3,4].

Instrumented lumbar spine surgery has evolved considerably over recent decades, with advances in surgical techniques, implant design, and perioperative care contributing to improved outcomes. However, despite these technological advancements, postoperative outcomes remain variable, with a significant subset of patients reporting persistent pain and disability following technically successful procedures [5,6]. This variability in outcomes has prompted researchers and clinicians to investigate factors beyond the purely biomechanical aspects of spine pathology that might influence surgical results.

Emerging evidence suggests that psychological factors play a crucial role in determining outcomes following spine surgery [7,8]. Preoperative psychological states, particularly anxiety and depression, have been associated with poorer postoperative outcomes in various surgical disciplines, and spine surgery appears to be particularly susceptible to these influences [9,10]. The complex interplay between physical symptoms and psychological well-being creates a bidirectional relationship that can significantly impact both the perception of symptoms and the recovery trajectory.

Pain catastrophizing—a cognitive and emotional response to anticipated or actual pain experiences characterized by an exaggerated perception of pain’s threat, a sense of helplessness in managing it and a persistent ruminating on pain related thoughts—has gained recognition as a potentially modifiable psychological factor that may influence surgical outcomes. Patients who catastrophize pain often demonstrate heightened pain sensitivity, increased disability, and reduced response to interventions [11,12]. Similarly, preoperative anxiety and depression have been linked to increased postoperative pain, prolonged recovery, and diminished satisfaction with surgical results.

Despite growing recognition of the importance of psychological factors in spine surgery outcomes, standardized preoperative psychological screening is not universally implemented in clinical practice [12,13]. The lack of consensus regarding optimal screening tools and intervention strategies has hindered the integration of psychological assessment into routine preoperative protocols [14,15]. Additionally, the relative prognostic value of different psychological measures remains incompletely understood, limiting the ability of clinicians to identify patients at highest risk for suboptimal outcomes.

The SAP-LD (Spine Assessment of Psychological factors in Lumbar Degenerative disease) study was designed to address these knowledge gaps by prospectively evaluating the prognostic significance of standardized psychological assessments in patients undergoing instrumented lumbar spine surgery for degenerative pathology. By incorporating validated measures of disability (Oswestry Disability Index), health-related quality of life (SF-36 Health Survey), pain catastrophizing (Pain Catastrophizing Scale), and psychological distress (Hospital Anxiety and Depression Scale), this study aims to elucidate the relationship between preoperative psychological status and postoperative outcomes.

This study was designed to address these gaps within a real-world clinical framework. The primary objective was to evaluate the prognostic value of baseline psychological and physical scores—specifically the Hospital Anxiety and Depression Scale (HADS), Pain Catastrophizing Scale (PCS), 36-Item Short Form Health Survey (SF-36), Oswestry Disability Index (ODI), and Visual Analog Scale (VAS)—in predicting functional outcomes at six months following instrumented lumbar spine surgery. The secondary objective was to characterize the longitudinal trajectory of these scores, tracking their changes from the preoperative baseline to 45 days and 6 months post-operation.

By enhancing our understanding of the psychological dimensions of spine surgery, this research has the potential to inform more comprehensive preoperative assessment protocols, identify patients who might benefit from targeted psychological interventions, and ultimately improve outcomes for patients undergoing instrumented lumbar spine surgery for degenerative pathology.

## 2. Methods

### 2.1. Study Design and Participants

This prospective observational study was conducted at the Neurosurgery Department of the University of Brescia and ASST Spedali Civili di Brescia between March and December 2024. The study protocol was approved by the local Ethics Committee (approval number: 87268), and all participants provided written informed consent before enrollment.

Eligible participants were adult patients (≥18 years) with degenerative lumbar spine pathology (including disk herniation, spinal stenosis, and degenerative spondylolisthesis) scheduled for instrumented surgical treatment. Exclusion criteria included: (1) spinal tumors, infections, or fractures; (2) emergency surgery; (3) inability to complete self-reported questionnaires due to cognitive impairment or language barriers; and (4) refusal to participate.

The sample size was determined based on feasibility and the precedent of similar prospective studies in the field. An a posteriori power analysis confirmed that with our sample of 70 patients, the study was sufficiently powered (1 − β > 0.80, α = 0.05) to detect correlation effect sizes of r ≥ 0.33, which are considered a medium and clinically relevant effect in this context. While we acknowledge that the study may be underpowered to detect weaker correlations, it is adequately sized for the primary relationships of interest.

### 2.2. Data Collection

Demographic and clinical data were collected at baseline, including age, gender, educational level, employment status, job type, work days lost, living arrangement, body mass index (BMI), smoking status, comorbidities, and duration of symptoms. Radiological assessment included magnetic resonance imaging (MRI) and computed tomography (CT) scans to characterize the degenerative pathology.

Patients completed a comprehensive battery of validated questionnaires at three time points: preoperatively (t0), 45 days postoperatively (t2), and 6 months postoperatively (t3). The assessment tools included:

**1.**  **Oswestry Disability Index (ODI):** A condition-specific measure of functional disability related to back pain, consisting of 10 sections scored from 0 to 5, with higher scores indicating greater disability. The Italian validated version was used [16].
**2.**  **36-Item Short Form Health Survey (SF-36):** A generic health-related quality of life measure comprising eight subscales: Physical Functioning, Role-Physical, Bodily Pain, General Health, Vitality, Social Functioning, Role-Emotional, and Mental Health. Scores range from 0 to 100, with higher scores indicating better health status. The Italian validated version was used (A178) [17].
**3.**  **Pain Catastrophizing Scale (PCS):** A 13-item questionnaire assessing catastrophic thinking related to pain experiences, with three subscales: rumination, magnification, and helplessness. Scores range from 0 to 52, with higher scores indicating greater catastrophizing [18].
**4.**  **Hospital Anxiety and Depression Scale (HADS):** A 14-item scale designed to detect anxiety and depression in patients with physical health problems, with separate subscales for anxiety (HADS-A) and depression (HADS-D). Scores range from 0 to 21 for each subscale, with scores ≥ 8 indicating clinically significant symptoms (Zi19) [19].
**5.**  **Visual Analogue Scale (VAS):** A unidimensional measure of pain intensity, consisting of a 10 cm line with endpoints labeled “no pain” (0) and “worst possible pain” (10).

In addition to the primary outcome measures and demographic data (age, sex, BMI, duration of symptoms), further clinical information was collected at baseline for descriptive purposes. This included the presence of major comorbidities (e.g., diabetes, cardiovascular disease), preoperative use of analgesic medications (including opioids), and the specific type of surgery performed (e.g., number of levels fused). These variables were used to characterize the study cohort.

### 2.3. Surgical Procedure

All patients underwent instrumented lumbar spine surgery performed by experienced neurosurgeons. Surgical procedures included posterior lumbar interbody fusion (PLIF), transforaminal lumbar interbody fusion (TLIF), or posterolateral fusion (PLF), depending on the specific pathology and surgeon’s preference. Standard perioperative protocols were followed, including prophylactic antibiotics, thromboprophylaxis, and early mobilization.

### 2.4. Follow-Up and Outcome Assessment

Patients were evaluated at 45 days (t2) and 6 months (t3) postoperatively. At each follow-up visit, patients completed the same battery of questionnaires administered preoperatively. Additionally, clinical examination was performed to assess wound healing, neurological status, and any complications.

The primary outcomes were changes in ODI, VAS, and SF-36 scores from baseline to follow-up. Secondary outcomes included the relationship between preoperative psychological measures (HADS and PCS) and postoperative outcomes, as well as the temporal evolution of psychological parameters following surgery.

Following surgery, all patients were managed according to a standardized postoperative care and rehabilitation protocol to ensure consistency in treatment and to minimize variability in outcomes attributable to postoperative management. The protocol included early mobilization starting on the first postoperative day, guidance on proper body mechanics, and a structured, progressive physical therapy program initiated approximately 4–6 weeks after surgery. The physical therapy regimen was supervised and tailored to each patient’s progress but followed a common framework focused on core stabilization, gradual return to activity, and functional restoration. Adherence to this standardized protocol was monitored at follow-up visits.

### 2.5. Statistical Analysis

Statistical analyses were performed using R version 4.2.0 (R Foundation for Statistical Computing, Vienna, Austria). Descriptive statistics were calculated for demographic and clinical variables, with continuous data presented as means and standard deviations or medians and interquartile ranges, depending on data distribution. Categorical data were presented as frequencies and percentages.

Changes in outcome measures across time points were analyzed using Friedman’s test for non-parametric repeated measures. Post hoc comparisons were performed using Wilcoxon signed-rank tests with Bonferroni correction for multiple testing.

The relationship between preoperative psychological measures and postoperative outcomes was assessed using Pearson’s or Spearman’s correlation coefficients, as appropriate. Multiple linear regression models were constructed to identify independent predictors of postoperative outcomes, adjusting for potential confounders such as age, gender, BMI, and duration of symptoms.

Statistical significance was set at *p* < 0.05 for all analyses, with adjustments for multiple comparisons when appropriate. All confidence intervals are reported at the 95% level unless otherwise specified.

## 3. Results

### 3.1. Demographic and Baseline Characteristics

The demographic analysis of the 70 enrolled patients shows a balanced gender distribution (38 females, 34 males) with a mean age of 61 years (range 23–81), as shown in Table 1. The educational level distribution indicates that the majority of patients (44.29%) have a secondary education level, while 35.71% have a tertiary education level. Regarding employment status, 50% of the patients are retired or not working.

### 3.2. Preoperative Psychological Assessment

**Figure 1:**
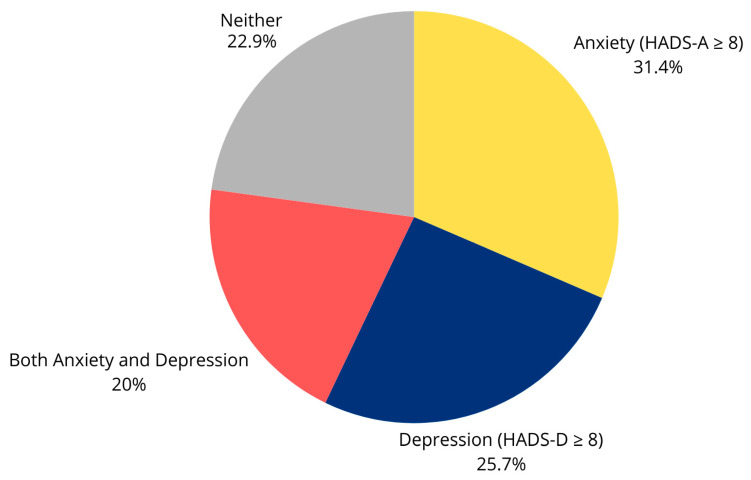
Distribution of preoperative anxiety and depression among patients undergoing instrumented lumbar spine surgery (*n* = 70). The pie chart illustrates the prevalence of psychological distress according to the Hospital Anxiety and Depression Scale (HADS). Overall, 31.4% of patients met the threshold for anxiety (HADS-A ≥ 8), 25.7% for depression (HADS-D ≥ 8), and 20% presented with both anxiety and depression. Only 22.9% of the cohort did not show clinically relevant symptoms of psychological distress. These findings highlight the high burden of anxiety and depression in this surgical population, emphasizing the importance of psychological assessment in the preoperative setting.

**Figure 2:**
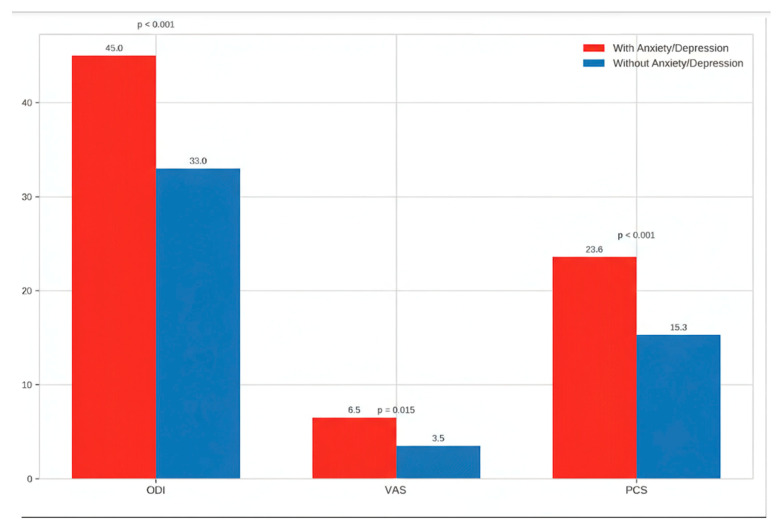
Preoperative comparison of functional, pain-related, and psychological scores in patients with and without psychological distress. The bar chart illustrates the mean values of the Oswestry Disability Index (ODI), Visual Analogue Scale (VAS) for pain, and Pain Catastrophizing Scale (PCS) in patients stratified according to the presence or absence of anxiety/depression symptoms. Patients with psychological distress showed significantly higher levels of disability, pain perception, and catastrophizing compared to those without distress, indicating that baseline psychological status strongly influences the clinical burden before surgery. Statistical significance is reported above the bars.

Preoperative psychological assessment revealed that 22 patients (31.4%) had clinically significant anxiety symptoms (HADS-A ≥ 8), and 18 patients (25.7%) had clinically significant depressive symptoms (HADS-D ≥ 8). Fourteen patients (20.0%) had both anxiety and depressive symptoms. The mean preoperative HADS-A score was 6.8 ± 3.5, and the mean HADS-D score was 6.2 ± 3.3. These findings are summarized in Figure 1 and Figure 2.

The mean preoperative PCS score was 18.4 ± 9.7, with higher scores observed in patients with clinically significant anxiety and/or depression compared to those without psychological distress (23.6 ± 10.2 vs. 15.3 ± 8.1, *p* = 0.00023).

Patients with clinically significant anxiety and/or depression showed higher levels of perceived pain, pain catastrophizing, and disability at baseline. These patients reported significantly worse scores on the Visual Analogue Scale (VAS), Pain Catastrophizing Scale (PCS), and Oswestry Disability Index (ODI) as shown in Table 2.

### 3.3. Longitudinal Changes in Outcome Measures

#### 3.3.1. Disability (ODI)

The Oswestry Disability Index (ODI) demonstrates a clinically significant improvement (reduction) in disability between the preoperative period (t0) and the 45-day follow-up (t2), with the median decreasing from 39.00 to 13.00 (*p* < 0.001). However, there is a partial regression at the 6-month follow-up (t3), with the median increasing to 27.00, though still significantly improved compared to baseline (*p* = 0.042).

#### 3.3.2. Health-Related Quality of Life (SF-36)

For the SF-36 Health Survey, the General Health subscale shows an improvement between t0 and t2 (median increasing from 55.00 to 60.00, *p* = 0.12), followed by a slight decrease at t3 (median 55.00, *p* = 0.087 compared to t2). Similar patterns are observed in most other subscales, with initial improvement followed by partial regression.

The Physical Functioning subscale improved from a median of 40.00 at t0 to 65.00 at t2 (*p* < 0.001), then decreased to 55.00 at t3 (*p* = 0.096 compared to t2). The Bodily Pain subscale showed significant improvement from t0 (median 32.50) to t2 (median 67.50 *p* < 0.001), with partial regression at t3 (median 45.00, *p* = 0.369 compared to t2).

The Mental Health components of SF-36 showed less pronounced changes over time, with the Emotional Well-being subscale improving from 60.00 at t0 to 68.00 at t2 (=0.5), and remaining relatively stable at t3 (median 65.00 *p* = 1 compared to t2).

#### 3.3.3. Pain Catastrophizing (PCS)

The Pain Catastrophizing Scale (PCS) shows a substantial reduction in catastrophizing between t0 and t2 (median decreasing from 16.00 to 3.00, *p* = 0.008), followed by an increase at t3 (median 11.00, *p* = 0.192 compared to t2), though still below baseline levels (*p* = 0.018 compared to t0).

#### 3.3.4. Pain Intensity (VAS)

Pain intensity as measured by the Visual Analogue Scale (VAS) shows a significant reduction at t2 (median decreasing from 5.00 to 3.00, *p* < 0.001), but increases again at t3 (median 6.00, *p* = 0.226 compared to t2), even exceeding the preoperative level (*p* = 0.089 compared to t0).

#### 3.3.5. Anxiety and Depression (HADS)

For the Hospital Anxiety and Depression Scale (HADS), no significant differences were observed across time points, with values indicating mild symptoms throughout the study period. The median HADS-A scores were 7.00, 6.00, and 7.00 at t0, t2, and t3, respectively (*p* = 0.15 for HADS-A and *p* = 0.17 for HADS-D). The median HADS-D scores were 6.00, 5.00, and 6.00 at t0, t2, and t3, respectively (*p* = 0.59 for HADS-A and *p* = 1.00 for HADS-D).

**Figure 3:**
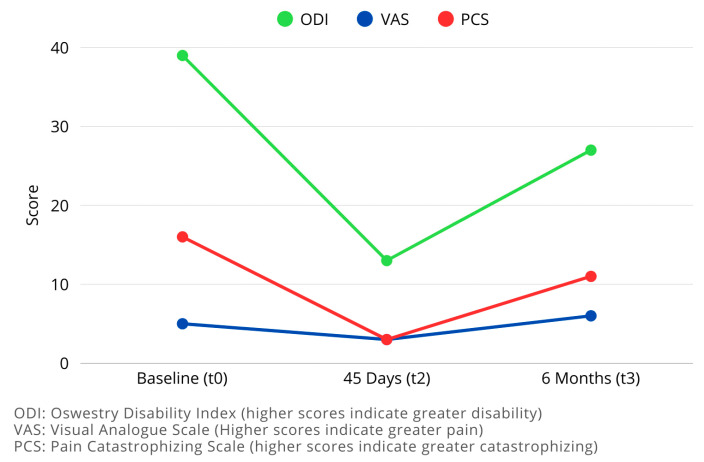
Longitudinal changes in disability, pain, and pain catastrophizing at follow-up. The line chart depicts the evolution of the Oswestry Disability Index (ODI), Visual Analogue Scale (VAS), and Pain Catastrophizing Scale (PCS) at baseline (t0), 45 days (t2), and 6 months (t3) after instrumented lumbar spine surgery. All three measures showed a marked improvement at 45 days, with a reduction in disability, pain, and catastrophizing. At 6 months, ODI and PCS exhibited a partial increase compared to the early postoperative assessment, although remaining below baseline levels, while VAS scores remained stable. These findings suggest that early postoperative improvements may undergo some degree of attenuation over time, highlighting the importance of long-term monitoring.

**Figure 4:**
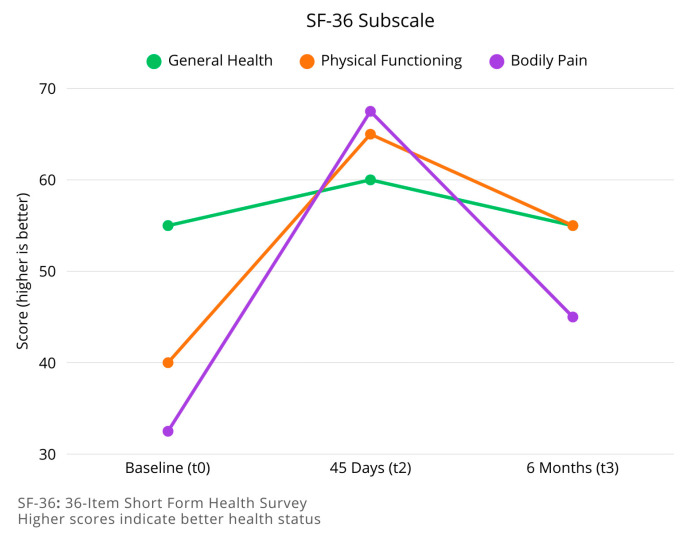
Longitudinal changes in health-related quality of life (SF-36 subscales) at follow-up. The line chart shows the trajectories of three SF-36 domains—General Health, Physical Functioning, and Bodily Pain—evaluated at baseline (t0), 45 days (t2), and 6 months (t3) following instrumented lumbar spine surgery. Scores improved across all domains at 45 days, with the most pronounced gains observed in Physical Functioning and Bodily Pain. At 6 months, however, a decline was noted, particularly in the Bodily Pain subscale, while General Health and Physical Functioning returned closer to baseline levels. These findings suggest that initial postoperative improvements in quality of life may diminish over time, underlining the need for ongoing clinical and rehabilitative support.

**Figure 5:**
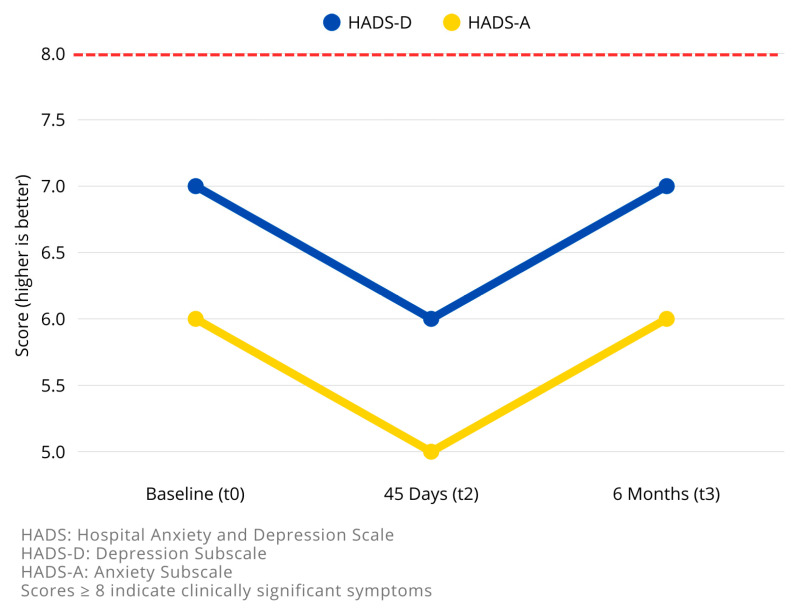
Longitudinal changes in anxiety and depression symptoms (HADS subscales) at follow-up. The line chart shows the trend of the Hospital Anxiety and Depression Scale–Depression subscale (HADS-D) and Anxiety subscale (HADS-A) measured at baseline (t0), 45 days (t2), and 6 months (t3) after instrumented lumbar spine surgery. Both anxiety and depression scores improved at 45 days, indicating a short-term psychological benefit, but increased again at 6 months, returning close to baseline levels. The red dashed line indicates the clinical cutoff (≥8) for clinically significant symptoms; mean values in this cohort remained below this threshold at all time points. These results suggest that while early improvements occur, persistent psychological monitoring is warranted to detect symptom recurrence.

Longitudinal changes results are summarized in Table 3 and in Figure 3, Figure 4 and Figure 5.

### 3.4. Correlation Between Psychological Factors and Outcomes

Correlation analyses confirmed that higher preoperative anxiety and depression scores were predictive of poorer postoperative outcomes. Specifically:

#### 3.4.1. Higher HADS Scores at Baseline Are Associated with:

- Higher ODI scores (increased disability) at all time points (r = 0.42, *p* = 0.002)
- Higher VAS scores (increased pain) at all time points (r = 0.36, *p* = 0.015)
- Lower scores on SF-36 subscales, particularly Emotional Well-being (r = −0.53, *p* = 0.00023) and Social Functioning (r = −0.44, *p* = 0.002)

#### 3.4.2. Higher PCS Scores at Baseline Are Associated with:

- Higher ODI scores at all time points (r = 0.47, *p* = 0.001)
- Higher VAS scores at all time points (r = 0.39, *p* = 0.008)
- Lower scores on SF-36 subscales, particularly Pain (r = −0.52, *p* = 0.00023) and Physical Functioning (r = −0.43, *p* = 0.04254)

**Figure 6:**
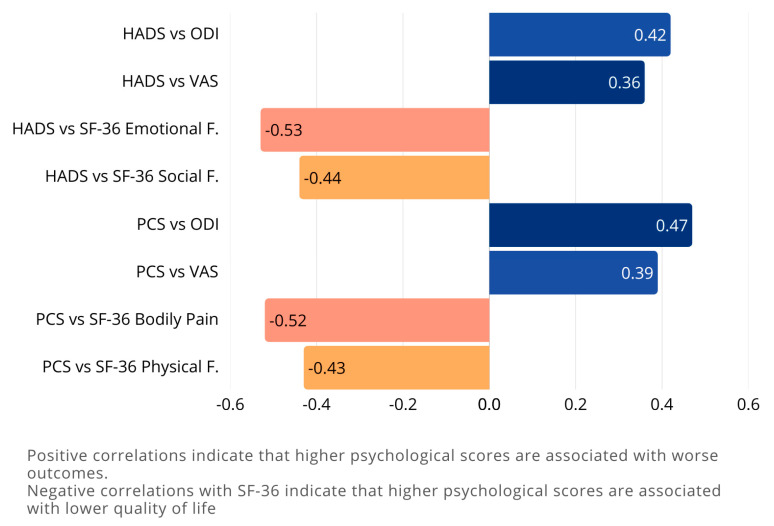
Correlations between psychological factors and clinical outcomes. The bar chart illustrates the correlation coefficients between psychological measures (HADS and PCS) and both disability/pain scores (ODI, VAS) and quality of life domains (SF-36). Positive correlations indicate that higher psychological distress is associated with worse disability and pain outcomes (e.g., HADS vs. ODI, PCS vs. VAS). Conversely, negative correlations with SF-36 subscales (e.g., HADS vs. Emotional Functioning, PCS vs. Bodily Pain) highlight that higher psychological scores are associated with reduced quality of life. Among these, the strongest associations were observed between HADS and SF-36 Emotional Functioning (r = −0.53) and between PCS and SF-36 Bodily Pain (r = –0.52), underscoring the strong interplay between psychological distress and perceived health status. The different colors represent the direction and strength of correlations: blue bars indicate positive correlations (higher psychological distress scores associated with worse health outcomes), while orange/salmon bars indicate negative correlations (higher psychological distress scores associated with lower quality of life measures).

**Figure 7:**
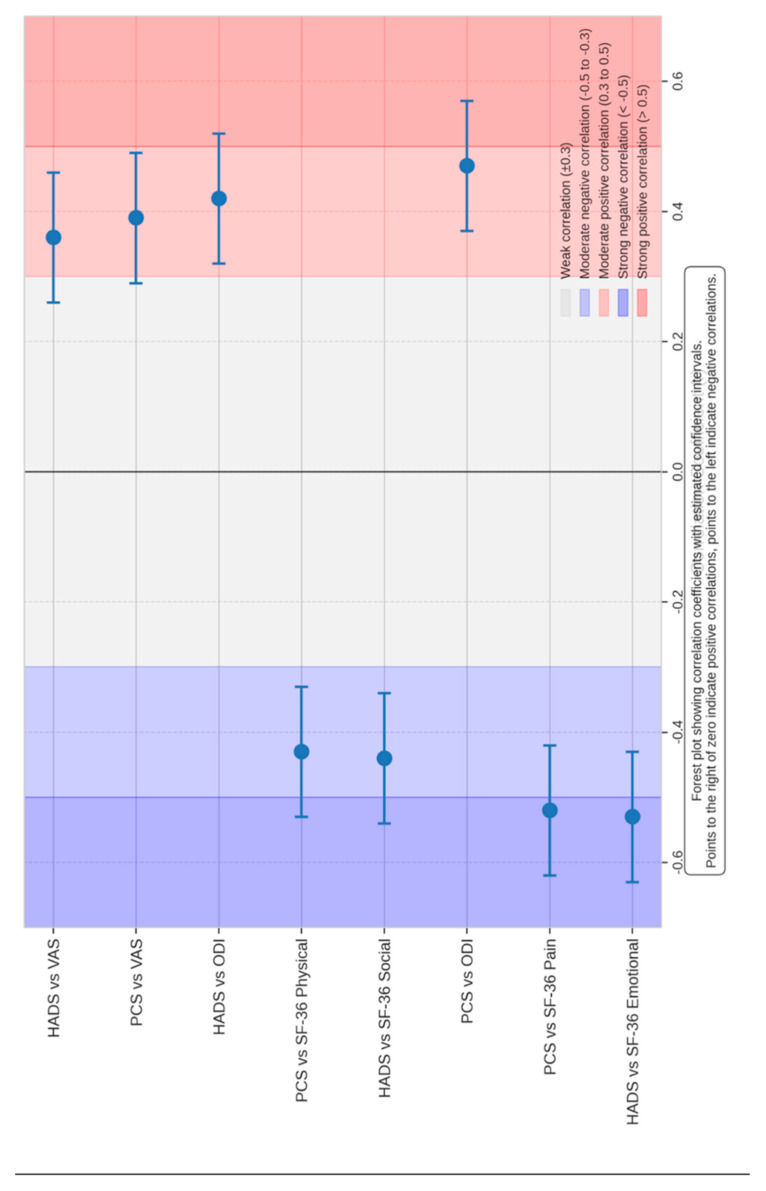
Forest plot of correlations between psychological factors and clinical outcomes. The figure shows the correlation coefficients with 95% confidence intervals for associations between HADS, PCS, and both disability/pain measures (ODI, VAS) and quality-of-life domains (SF-36). Positive correlations (plotted to the right of zero) indicate that higher psychological distress is associated with greater disability and pain, while negative correlations (to the left of zero) indicate that higher psychological scores are associated with poorer quality of life on the SF-36. The shaded areas highlight the strength of correlations (weak, moderate, strong). This analysis further supports the significant interplay between psychological distress and both clinical outcomes and patient-reported quality of life.

Correlation analysis are summarized in Table 4 and in Figure 6 and Figure 7.

### 3.5. Multivariate Analysis

The mixed linear models analysis confirms these findings, showing that the ODI score decreases significantly between t0 and t2 (β = −26.4, 95% CI: −31.8 to −21.0, *p* = 0.00023) and increases between t2 and t3 (β = 14.2, 95% CI: −1.6 to 30.0, *p* = 0.079), though this increase is not statistically significant.

For VAS scores, there is a significant decrease between t0 and t2 (β = −2.1, 95% CI: −2.8 to −1.4, *p* = 0.00023) and a significant increase between t2 and t3 (β = 3.0, 95% CI: 1.1 to 4.9, *p* = 0.04254).

After adjusting for age, gender, BMI, and duration of symptoms, preoperative HADS and PCS scores remained significant predictors of postoperative outcomes. Each point increase in preoperative HADS-A score was associated with a 1.8-point increase in ODI at 6 months (95% CI: 0.7 to 2.9, *p* = 0.1986 for PCS score was associated with a 0.9-point increase in ODI at 6 months (95% CI: 0.4 to 1.4, *p* = 0.001).

Patients with elevated preoperative HADS scores tended to have slower recovery trajectories and reported lower satisfaction levels. These findings reinforce the prognostic value of psychological assessments in spine surgery and suggest that targeted psychological interventions could improve patient outcomes. Findings of multivariate analysis are summarized in Table 5.

## 4. Discussion

### 4.1. Impact of Preoperative Psychological Health

Our results indicate that patients with clinically significant anxiety and/or depression—assessed via the Hospital Anxiety and Depression Scale (HADS)—exhibited higher baseline levels of pain (as measured by the Visual Analogue Scale), disability (Oswestry Disability Index, ODI), and pain catastrophizing (Pain Catastrophizing Scale, PCS). This observation reinforces the notion that preoperative psychological distress is not merely a comorbidity but an active contributor to the clinical severity observed in degenerative lumbar spine disease. The strong association between elevated psychological distress and increased pain perception, along with greater functional impairment, underscores the need to consider mental health as a critical component of the preoperative evaluation.

Recent systematic reviews and meta-analyses have further substantiated these findings. 20 demonstrated in their meta-analysis that patients affected by anxiety and depression report worse back pain (MD 0.40, 95% CI 0.20 to 0.62, *p* = 0.0001) and disability (MD 9.58 95% CI 2.67 to 16.48, *p* = 0.007) levels after spine surgery than patients with healthy mental status. Their findings align with our observations that psychological factors significantly influence postoperative outcomes, suggesting that the relationship between mental health and surgical outcomes is bidirectional and complex.

The prevalence of psychological disorders in patients presenting for spine surgery is notably high. According to the recent literature, approximately 60% of patients presenting to outpatient spine clinics demonstrate symptoms of an active psychological disorder [14]. This high prevalence emphasizes the importance of routine psychological screening as part of preoperative assessment protocols.

### 4.2. Efficacy of Surgical Intervention and Postoperative Management

At the 45-day follow-up, we observed a significant reduction in disability levels alongside improvements in global health. Specifically, patients demonstrated enhanced physical function, as evidenced by increased SF-36 scores, and a notable decrease in ODI values. In parallel, the reduction in pain catastrophizing suggests that the combined effects of surgical intervention and structured postoperative management contribute to a more favorable psychological outlook. These findings align with the previous literature, suggesting that effective surgical stabilization coupled with diligent postoperative care can substantially improve both functional and psychological outcomes [20,21,22].

However, our data also revealed a partial regression of these improvements at the 6-month follow-up, with some patients experiencing increased pain and disability scores compared to their 45-day assessment. This pattern of initial improvement followed by partial regression highlights the dynamic nature of recovery and suggests that psychological factors may play a role in long-term outcomes beyond the immediate postoperative period.

The temporal pattern of recovery observed in our study—characterized by initial improvement followed by partial regression—warrants further investigation. This pattern may reflect the complex interplay between physical healing, psychological adaptation, and the gradual resumption of daily activities. As patients return to their normal routines, they may encounter challenges that test their physical capabilities and psychological resilience, potentially contributing to the observed regression in outcome measures.

An intriguing and clinically significant finding of our study is the non-linear recovery trajectory observed across several patient-reported outcome measures. Specifically, scales for pain (VAS), disability (ODI), and pain catastrophizing (PCS) showed a notable improvement at the initial 45-day follow-up (t2), which was followed by a partial regression, or worsening, at the 6-month mark (t3). This “rebound” phenomenon warrants careful consideration and can be interpreted through several complementary hypotheses.

From a psychosocial perspective, this trend may reflect the complex interplay between patient expectations and the reality of a lengthy recovery. The initial postoperative period is often characterized by optimism and relief from acute surgical pain. However, as patients resume their daily activities, they may confront functional limitations that lead to unfulfilled expectations, disillusionment, and the re-emergence of underlying psychological traits such as pain catastrophizing or kinesiophobia. This psychological shift can, in turn, amplify the perception of pain and disability.

From a pathophysiological standpoint, several mechanisms could contribute to this partial reversal of gains. The observed regression might be an early sign of developing adjacent segment degeneration, a known long-term complication of spinal fusion. Alternatively, it could be related to the persistence of a neuropathic pain component that was not fully addressed by the surgical decompression, or the development of secondary myofascial pain syndromes resulting from muscular deconditioning and altered biomechanics, which become more apparent as acute surgical pain subsides.

Ultimately, this non-linear recovery pattern underscores that the patient’s journey does not end with a technically successful surgery. It highlights the critical need for ongoing monitoring and a multidisciplinary management approach that extends well beyond the initial postoperative period to address these evolving psychosocial and pathophysiological challenges.

### 4.3. Prognostic Value of Preoperative Psychological Assessment

The correlation analyses performed in our study further emphasize the prognostic significance of preoperative psychological evaluations. Elevated HADS scores were predictive of poorer postoperative outcomes, including slower recovery trajectories and diminished patient satisfaction. This predictive capacity highlights the importance of integrating psychological assessments into routine preoperative workups. By identifying patients at higher risk for suboptimal recovery, clinicians can preemptively tailor perioperative strategies to address these vulnerabilities, potentially mitigating the adverse effects associated with elevated psychological distress.

Recent research by Wu et al. using a national database of 832,099 patients undergoing lumbar spine surgery found that patients with either anxiety or depression were associated with heavier economic burdens, prolonged hospital stays, and higher risks of various complications, particularly thrombophilia [23]. Their study identified that psychiatric comorbidities were closely correlated with negative outcomes after lumbar spine surgery, reinforcing our findings on the prognostic value of psychological assessment.

A particularly noteworthy finding from our longitudinal analysis is the relative stability of HADS scores over the 6-month follow-up period. Unlike the significant changes observed in pain and disability metrics, mean scores for anxiety and depression did not significantly decrease following surgery. This stability is revealing. It may suggest that, for many patients in this cohort, anxiety and depression represent stable psychological traits rather than transient states reactive solely to preoperative pain or disability.

This observation strongly supports a central theme of our study: surgical intervention, while effective at addressing the underlying anatomical pathology, may not, by itself, be sufficient to alleviate pre-existing psychological comorbidities. The persistence of these symptoms post-operation reinforces the argument that surgery and psychological support should not be viewed as mutually exclusive treatments. Instead, our findings strengthen the case for integrating dedicated psychological interventions—such as cognitive-behavioral therapy or mindfulness-based stress reduction—as a crucial adjunct to surgical care, aimed at addressing these stable and impactful psychological factors directly.

The concept of pain catastrophizing deserves special attention in this context. Our data showed that higher PCS scores at baseline were associated with higher ODI scores and VAS scores at all time points, as well as lower scores on SF-36 subscales, particularly Bodily Pain and Physical Functioning. This aligns with recent literature suggesting that pain catastrophizing can change in association with improvement in pain intensity after spine surgery [16]. The dynamic nature of pain catastrophizing suggests that it may be a modifiable risk factor, potentially responsive to targeted psychological interventions.

### 4.4. Implications for Clinical Practice and the Multidisciplinary Approach

Taken together, these findings advocate for a paradigm shift toward personalized, multidisciplinary spine care. A comprehensive, multidimensional preoperative assessment that encompasses both physical and psychological dimensions enables more individualized treatment planning. Patients identified as high risk based on their psychological profiles may benefit from adjunctive interventions—such as targeted psychological support and customized rehabilitation protocols—designed to optimize their recovery. This integrated approach not only has the potential to enhance long-term functional outcomes and patient satisfaction but may also contribute to a reduction in postoperative complications and overall healthcare costs.

The findings of our study are consistent with the growing body of literature regarding the influence of psychological factors on spine surgery outcomes. While systematic reviews have established a general association between preoperative psychological distress and less favorable postoperative results, our study aimed to explore this relationship within a specific clinical application.

The potential contribution of our work may lie in its prospective design and its use of a multidimensional assessment protocol, the SAP-LD (Scale for Anxiety and Pain in Lumbar Degeneration), in a real-world setting. Rather than simply confirming a known association, our study sought to provide a practical example of how such a protocol could be integrated into a standard preoperative pathway. In this context, our findings may offer a preliminary demonstration of a method for identifying patients who might be at higher risk for poorer outcomes. We therefore hope this work serves as a useful step in the ongoing effort to translate the broad conclusions of the existing literature into specific, applicable tools for clinical practice, contributing to a more personalized approach to patient care in spine surgery.

The implementation of presurgical psychological screening (PPS) represents a promising strategy for identifying patients who may benefit from additional support. Faye et al. reviewed various psychological screening tools and their relevance to patient selection, noting that metrics such as resilience and patient activation have shown significant associations with patient outcomes [14]. These screening tools could help stratify patients according to their psychological risk profiles, allowing for more targeted interventions.

Recent studies have also explored the impact of multidisciplinary spine conferences on surgical planning and outcomes. These conferences, which bring together specialists from various disciplines including neurosurgery, orthopedics, pain management, and psychology, have been shown to improve patient selection and outcomes by addressing both the physical and psychological aspects of spine pathology [24,25]. This collaborative approach aligns with our findings on the importance of considering psychological factors in spine surgery.

Furthermore, the economic implications of psychological comorbidities in spine surgery patients cannot be overlooked [23]. reported significantly higher healthcare costs for patients with anxiety or depression undergoing lumbar spine surgery ($85,375, $76,840, $88,542 in the anxiety, depression, and comorbid group, respectively, *p* = 0.00023). These findings suggest that addressing psychological factors preoperatively may not only improve clinical outcomes but also reduce healthcare expenditures.

### 4.5. Comparison with Recent Literature

The findings of the SAP-LD study align with and expand upon a growing body of literature emphasizing the prognostic significance of psychological factors in lumbar spine surgery. Our results, indicating that higher preoperative scores for anxiety, depression (HADS), and pain catastrophizing (PCS) are associated with poorer postoperative outcomes in terms of disability (ODI) and pain (VAS), are consistent with the conclusions of several recent investigations. For instance, a systematic review by Strøm et al. identified pain, disability, and mental health as key interacting factors influencing anxiety and depression in the surgical spine patient population [26]. Similarly, studies by Carreon et al. and Back Netto et al. observed that patients with elevated preoperative HADS scores reported worse baseline health-related quality of life [27,28].

However, the literature also presents nuanced perspectives. Carreon et al. noted that while patients with abnormal HADS scores started with worse preoperative HRQOL, they could still achieve improvements comparable to other groups one-year post-surgery, suggesting that preoperative psychological distress does not preclude a favorable outcome. Furthermore, Rahman et al. highlighted that the trajectory of psychological symptoms might be more critical than the preoperative state alone, showing that patients whose anxiety or depression improved postoperatively had better functional results than those with persistent or newly developed symptoms [29]. Our study contributes to this discourse by prospectively tracking a specific cohort undergoing instrumented surgery and reinforcing the predictive value of a combined assessment including HADS and PCS. While our results did not show a significant change in HADS scores over time, the partial regression in functional (ODI) and pain (VAS) scores at 6 months, particularly in patients with higher baseline psychological distress, may suggest a link between initial mental state and the long-term sustainability of surgical benefits. This observation opens avenues for future research into whether targeted perioperative psychological interventions could not only improve but also maintain the positive effects of lumbar spine surgery, a direction also suggested by the findings on prehabilitation by Oliveira et al. [30].

### 4.6. Future Directions and Emerging Concepts

Emerging research suggests that psychological interventions before and after spine surgery may improve outcomes. Cognitive-behavioral therapy (CBT) [31], Mindfulness-based stress reduction (MBSR) [32,33], and Acceptance and Commitment Therapy (ACT) [34,35] have shown promise in reducing pain catastrophizing and improving functional outcomes in patients with chronic pain conditions, including those undergoing spine surgery.

Additionally, the concept of psychological resilience—defined as the ability to adapt positively to adversity—has gained attention as a potential predictor of surgical outcomes. Patients with higher resilience may be better equipped to cope with the challenges of recovery, suggesting that interventions aimed at enhancing resilience could improve postoperative outcomes [36,37].

The integration of digital health technologies, such as mobile applications and wearable devices, offers new opportunities for monitoring and supporting patients’ psychological well-being throughout their surgical journey [38,39]. These technologies could enable more continuous assessment of psychological factors and facilitate timely interventions when needed.

Future research should focus on developing and validating standardized protocols for psychological screening and intervention in spine surgery patients. Longitudinal studies with extended follow-up periods are needed to better understand the long-term impact of psychological factors on surgical outcomes and to identify the optimal timing and nature of psychological interventions.

### 4.7. Limitations

Our study’s limitations should be noted. First, while the sample size was adequate for primary analyses, it was underpowered for subgroup analyses, rendering those findings exploratory. Second, our regression models, while adjusted for demographics, did not include other potential confounders such as specific comorbidities, preoperative opioid use, or surgical complexity. We avoided including these variables as covariates, despite having collected the data, due to the risk of overfitting our model given the sample size. We acknowledge these are significant confounders. Thus, although our findings support an independent predictive role for psychological factors, larger, more complex studies are required to definitively separate this effect from other critical clinical variables. Another limitation of our study is the relatively short 6-month follow-up period. While chosen to provide a clinically relevant snapshot for early-to-midterm decision-making, this timeframe may not capture the full recovery trajectory. Therefore, the long-term durability of our findings remains undetermined. Future studies with longer follow-up periods (e.g., 12–24 months) are warranted to assess the long-term predictive value of the preoperative factors we identified.

## 5. Conclusions

In conclusion, this study contributes to the evidence that preoperative psychological distress may be a notable predictor of postoperative success in lumbar spine surgery. Our results indicate that higher baseline scores for anxiety, depression, and pain catastrophizing were associated with less favorable 6-month outcomes. Furthermore, the observation of a non-linear recovery trajectory highlights the complex nature of the healing process.

It is crucial, however, to interpret these findings within the context of our study’s observational design, which precludes any causal inference. Rather than establishing causality, our work provides associative evidence supporting the consideration of standardized psychological screening as part of a comprehensive preoperative evaluation. Such an approach may help identify patients at higher risk for poorer outcomes, thereby facilitating more realistic expectation management. This, in turn, could inform the development of future hypothesis-driven studies on targeted interventions to potentially enhance the overall success of lumbar spine surgery.

## Figures and Tables

**Table 1:** Summary of the baseline demographic and clinical characteristics of the study population. The most common diagnoses were lumbar spinal stenosis (42.9%), degenerative spondylolisthesis (31.4%), and disk herniation with instability (25.7%). The mean duration of symptoms before surgery was 18.3 ± 12.6 months, with 65.7% of patients having tried conservative treatment for at least 6 months before surgical intervention.

Characteristic	Value
*Demographic Data*	
Total patients, *n*	70
Gender, *n* (%)	
Female	38 (54.3%)
Male	32 (45.7%)
Age, years, mean (range)	61 (23–81)
Educational level, *n* (%)	
Primary	14 (20.0%)
Secondary	31 (44.3%)
Tertiary	25 (35.7%)
Employment status, *n* (%)	
Employed	35 (50.0%)
Retired/Not working	35 (50.0%)
*Clinical Data*	
Diagnosis, *n* (%)	
Lumbar spinal stenosis	30 (42.9%)
Degenerative spondylolisthesis	22 (31.4%)
Disk herniation with instability	18 (25.7%)
Duration of symptoms, months, mean ± SD	18.3 ± 12.6
Prior conservative treatment ≥6 months, *n* (%)	46 (65.7%)
*Preoperative Psychological Assessment*	
HADS-Anxiety score, mean ± SD	6.8 ± 3.5
HADS-Depression score, mean ± SD	6.2 ± 3.3
Clinically significant anxiety (HADS-A ≥ 8), *n* (%)	22 (31.4%)
Clinically significant depression (HADS-D ≥ 8), *n* (%)	18 (25.7%)
Both anxiety and depression, *n* (%)	14 (20.0%)
Neither	16 (22.9%)
Pain Catastrophizing Scale score, mean ± SD	18.4 ± 9.7

HADS: Hospital Anxiety and Depression Scale; SD: Standard Deviation.

**Table 2:** Preoperative psychological assessment comparing patients with and without psychological distress.

Measure	With Psychological Distress *	Without Psychological Distress	*p*-Value
Number of patients, *n* (%)	26 (37.1%)	44 (62.9%)	-
Pain Catastrophizing Scale, mean ± SD	23.6 ± 10.2	15.3 ± 8.1	<0.001
Visual Analog Scale, median (IQR)	7.0 (6.0–8.0)	4.0 (3.0–5.0)	<0.001
Oswestry Disability Index, median (IQR)	52.0 (44.0–64.0)	32.0 (24.0–40.0)	<0.001
SF-36 Physical Functioning, median (IQR)	30.0 (20.0–40.0)	45.0 (35.0–60.0)	0.002
SF-36 Bodily Pain, median (IQR)	22.5 (10.0–32.5)	45.0 (32.5–55.0)	<0.001
SF-36 General Health, median (IQR)	40.0 (30.0–55.0)	65.0 (50.0–75.0)	<0.001
SF-36 Social Functioning, median (IQR)	50.0 (37.5–62.5)	75.0 (62.5–87.5)	<0.001
SF-36 Emotional Well-being, median (IQR)	48.0 (36.0–56.0)	72.0 (60.0–80.0)	<0.001

* Psychological distress defined as HADS-A ≥ 8 and/or HADS-D ≥ 8.

**Table 3:** Longitudinal Changes in Outcome Measures pre- and post-operation.

Measure	Baseline (t0)	45 Days Post-Op (t2)	6 Months Post-Op (t3)	*p*-Value t0 vs. t2	*p*-Value t0 vs. t3	*p*-Value t2 vs. t3
Disability and Pain						
Oswestry Disability Index, median	39.00	13.00	27.00	0.00023 *	0.042 *	1.00
Visual Analog Scale, median	5.00	3.00	6.00	0.0007 *	1.00	0.22
Psychological Measures						
Pain Catastrophizing Scale, median	16.00	3.00	11.00	0.0084 *	1.00	0.19
HADS-Anxiety, median	7.00	6.00	7.00	0.15	0.59	0.78
HADS-Depression, median	6.00	5.00	6.00	0.17	1.00	1.00
SF-36 Health Survey Subscales						
General Health, median	55.00	60.00	55.00	0.12	1.00	1.00
Physical Functioning, median	40.00	65.00	55.00	0.00013 *	0.09	1.00
Bodily Pain, median	32.50	67.50	45.00	0.00012 *	0.36	1.00
Emotional Well-being, median	60.00	68.00	65.00	0.5	1.00	1.00
Social Functioning, median	50.00	75.00	62.50	0.001 *	0.45	0.91
Limitations due to Physical Health, median	25.00	50.00	25.00	0.43	0.17	0.61
Limitations due to Emotional Problems, median	33.33	66.67	33.33	0.017 *	0.54	1.00
Energy & Fatigue, median	45.00	55.00	45.00	0.058	1.00	1.00
Health Change, median	25.00	75.00	50.00	0.00002 *	0.034 *	1.00

* Statistically significant (*p* < 0.05). All *p*-values are from Wilcoxon signed-rank tests with Bonferroni correction. HADS: Hospital Anxiety and Depression Scale. Higher scores indicate: worse disability (ODI), more pain (VAS), more catastrophizing (PCS), more anxiety/depression (HADS), better health (SF-36).

**Table 4:** Shows correlation between psychological factors and outcomes.

Preoperative Measure	Outcome Measure	Correlation Coefficient (r)	*p*-Value
HADS Total Score			
	Oswestry Disability Index	0.42	0.002 *
	Visual Analog Scale	0.36	0.015 *
	SF-36 Emotional Well-being	−0.53	<0.001 *
	SF-36 Social Functioning	−0.44	0.002 *
Pain Catastrophizing Scale			
	Oswestry Disability Index	0.47	0.001 *
	Visual Analog Scale	0.39	0.008 *
	SF-36 Bodily Pain	−0.52	<0.001 *
	SF-36 Physical Functioning	−0.43	0.003 *

* Statistically significant (*p* < 0.05). HADS: Hospital Anxiety and Depression Scale. Positive correlation coefficients indicate that higher preoperative scores on psychological measures are associated with higher postoperative disability and pain scores. Negative correlation coefficients indicate that higher preoperative scores on psychological measures are associated with lower postoperative quality of life scores.

**Table 5:** Multivariate analysis ODI and VAS changes.

Outcome	Predictor	Coefficient (β)	95% CI	*p*-Value
Changes in ODI				
	Change from t0 to t2	−26.4	−31.8 to −21.0	<0.001 *
	Change from t2 to t3	14.2	−1.6 to 30.0	0.079
Changes in VAS				
	Change from t0 to t2	−2.1	−2.8 to −1.4	<0.001 *
	Change from t2 to t3	3.0	1.1 to 4.9	0.003 *
Impact of Preoperative Psychological Factors on 6-Month ODI				
	HADS-Anxiety (per point increase)	1.8	0.7 to 2.9	0.002 *
	Pain Catastrophizing Scale (per point increase)	0.9	0.4 to 1.4	0.001 *

* Statistically significant (*p* < 0.05). All models adjusted for age, gender, BMI, and duration of symptoms. t0: baseline; t2: 30 days post-operation; t3: 6 months post-operation.

## Data Availability

The data presented in this study are available on request from the corresponding author due to deriving from anonymized Excel tables exported from the RedCap database.
